# Carbon black and titanium dioxide nanoparticles elicit distinct apoptotic pathways in bronchial epithelial cells

**DOI:** 10.1186/1743-8977-7-10

**Published:** 2010-04-16

**Authors:** Salik Hussain, Leen CJ Thomassen, Ioana Ferecatu, Marie-Caroline Borot, Karine Andreau, Johan A Martens, Jocelyne Fleury, Armelle Baeza-Squiban, Francelyne Marano, Sonja Boland

**Affiliations:** 1 Université Paris Diderot - Paris 7, Unit of Functional and Adaptive Biology (BFA) CNRS EAC 4413, Laboratory of Molecular and Cellular Responses to Xenobiotics, 75205 Paris, France; 2 Department of Pathology, University of Veterinary and Animal Sciences, Lahore, Pakistan; 3 Center for Surface Chemistry & Catalysis, Katholieke Universiteit Leuven, Kasteelpark Arenberg 23, 3001 Heverlee, Belgium; 4 INSERM, Unité 955, 94000 Créteil, France

## Abstract

**Background:**

Increasing environmental and occupational exposures to nanoparticles (NPs) warrant deeper insight into the toxicological mechanisms induced by these materials. The present study was designed to characterize the cell death induced by carbon black (CB) and titanium dioxide (TiO_2_) NPs in bronchial epithelial cells (16HBE14o- cell line and primary cells) and to investigate the implicated molecular pathways.

**Results:**

Detailed time course studies revealed that both CB (13 nm) and TiO_2_(15 nm) NP exposed cells exhibit typical morphological (decreased cell size, membrane blebbing, peripheral chromatin condensation, apoptotic body formation) and biochemical (caspase activation and DNA fragmentation) features of apoptotic cell death. A decrease in mitochondrial membrane potential, activation of Bax and release of cytochrome *c *from mitochondria were only observed in case of CB NPs whereas lipid peroxidation, lysosomal membrane destabilization and cathepsin B release were observed during the apoptotic process induced by TiO_2 _NPs. Furthermore, ROS production was observed after exposure to CB and TiO_2 _but hydrogen peroxide (H_2_O_2_) production was only involved in apoptosis induction by CB NPs.

**Conclusions:**

Both CB and TiO_2 _NPs induce apoptotic cell death in bronchial epithelial cells. CB NPs induce apoptosis by a ROS dependent mitochondrial pathway whereas TiO_2 _NPs induce cell death through lysosomal membrane destabilization and lipid peroxidation. Although the final outcome is similar (apoptosis), the molecular pathways activated by NPs differ depending upon the chemical nature of the NPs.

## Background

Nanotechnology industry is expanding at a rapid rate but in-depth exploration of the health and environmental effects of these materials is still warranted[1]. There is increasing evidence linking the NPs with human health problems. It has already been shown that inhaled carbonaceous NPs possess the potential to aggravate existing respiratory disorders, such as asthma or bronchitis[2,3]. Translocation of NPs from the lungs towards other organs has been demonstrated and possible consequences include inflammation, heart rate and function anomalies, homeostatic disturbances and oxidative stress[4,5]. More recently it has also been shown that pre-injected titanium dioxide nanoparticles can transform benign cells into aggressive metastatic tumor cells[6]. On the basis of current knowledge, there is increasing need for the risk assessment of both CB and TiO_2 _due to increased environmental and occupational exposures. CB and TiO_2 _are among the most abundantly produced and widely utilized NPs. Major sources of CB NPs include combustion (considered as combustion derived ultrafine particles) and industry. These particles also represent the core of atmospheric pollution particles. TiO_2 _NPs are used in the preparation of sunscreens, cosmetics and tooth pastes[7,8]. Some recent estimates of annual global nano TiO_2 _production range between 5000-6400 metric tones[9,10]. These enormous amounts of nanomaterial produced raise the possibilities of occupational and environmental exposures.

NP exposures can lead to disturbances in the cellular homeostatic mechanisms resulting either in adaptive cellular responses or cell death. NP-induced perturbations of cellular mechanisms might act as basis of different pathophysiological processes depending upon the concentration and duration of exposure[11]. Cell death could occur either by an abrupt process named necrosis or by a tightly regulated or programmed process (apoptosis and autophagy). Necrotic cell death occurs in different human pathologies like cerebral ischemia, myocardial infarction and acute organ failures. Apoptosis is a key event in many physiological, biochemical as well as pathological phenomenon. Either an excess or a reduced apoptotic process is involved in many pathological conditions such as autoimmune diseases, neurodegenerative disorders and carcinogenesis[12]. Apoptosis plays an important role in the pathogenesis of different respiratory disorders such as asthma, emphysema, and acute respiratory distress syndrome[13,14].

Reactive oxygen species (ROS) play a dual role in the fate of cell i.e. causing cell death as well as acting as second messengers to induce an adaptive cell response[15]. Indeed, oxidative stress has been shown to induce cell death by a variety of mechanisms [16-18]. A hierarchical model for NP toxicity also describes the possibility of higher oxidative stress levels leading to cell death induction[11]. Different types of NPs have been shown to induce oxidative stress [19-21] but the role of oxidative stress in NP induced cell death has not yet been completely elaborated.

This work was carried out on human bronchial epithelial cells (16HBE14o- cell line and normal human bronchial epithelial cells) which represent one of the target cells at the portal of entry of NPs in human body. The present study was designed to investigate the molecular mechanism/pathways implicated in the cell death induced by two chemically distinct but nearly same sized NPs (CB and TiO_2_). Furthermore, the involvement of oxidative stress in cell death induction by NPs was also investigated. To the best of our knowledge, this is the first report elaborating the molecular pathways involved in apoptosis induction by CB and TiO_2 _NPs showing that distinct cellular mechanisms are involved in apoptosis induction by these two NPs.

## Results

### NP Characterization

**Figure 1:**
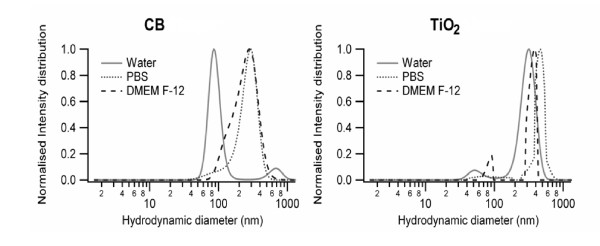
**Size distributions of NPs in suspension**. Dynamic light scattering (DLS) analysis of CB and TiO_2 _NPs after suspending in water, PBS or DMEM-F12 cell culture media was performed to determine size distributions and hydrodynamic diameters of NPs.

Before cell culture experimentation, NPs were characterized for their behavior in different media. Detailed physico-chemical characteristics (diameter, surface area, zeta potentials and hydrodynamic diameters of the suspended particles in water, PBS and DMEM F-12) of the NPs were determined and are given in Table 1. To this end extent of the aggregation of NPs was determined with the help of DLS and electron microscopy. Distribution curves show variable degree of aggregation for both NPs in all the media with larger size of aggregates in DMEM F-12. CB has a large population of small aggregates (86 nm) in water, whereas in PBS and DMEM F-12 the dispersion of CB is heterogeneous with an average size of respectively 281 and 253 nm. For the titanium dioxide sample, small aggregates were present in the water and DMEM F-12 suspension, but in all media the size distribution was dominated by larger (300-500 nm) aggregates (Figure 1). CB and TiO_2 _NPs have a negative surface charge in water, which is stabilizing the suspensions via repulsive forces. In physiological solutions (PBS and DMEM F-12), the surface charges were systematically less negative.

**Table 1:** Physico-chemical characteristics of Carbon black and Titanium dioxide nanoparticles

				Zeta potential (mV)			Hydrodynamic diameter (nm)				
	Size (ø) nm^a^	Average TEM Size^b^ (nm+sd)	BET surface area (m^2 ^. g^-1^)	Water	PBS	DMEM F-12	Water	PBS	DMEM F-12	Crystalline Structure	Impurities (% basis)
CB	13	23 ± 6	350	-48	-4	-7	86 and 668	281	253	amorphous	non^c^
TiO_2_	15	12 ± 2	210	-24	-11	-9	53 and 311	461	86 and 356	anatase	0.3^d^

^a ^Manufacturer's data

^b^Hussain et al 2009

^c ^PAH contents after 8 h toluene extraction (manufacturer's data)

^d ^Trace metal contents (manufacturer's data)

### Characterization of Cell Death Induced by NPs

**Figure 2:**
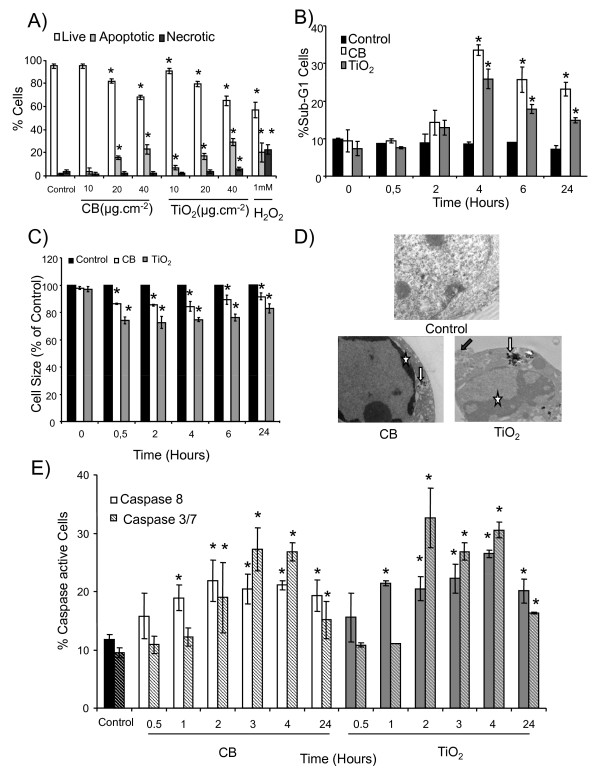
**Characterization of cell death induced by CB and TiO_2 _NPs in 16HBE14o- cells**. A) A dose response of FDA (fluorescein diacetate) and EtBr (ethidium bromide) staining after 4 hours of NP exposure (10-40 μg.cm^-2^) analyzed by fluorescence microscopy. 1 mM H_2_O_2_was used as positive control. B) Flow cytometry analysis of time course (0-24 h) of DNA fragmentation (% of cells in Sub-G1 peak). Cells were treated for the indicated durations with CB and TiO2 NPs (20 μg.cm^-2^), fixed and stained with propidium iodide for cell cycle analysis by flow cytometry. C) A time course study (0-24h) of the decrease in relative average cell size by measuring decrease in forward scatter (FS, % of control) of the laser in flow cytometry after NP exposures (20 μg.cm^-2^). D) Transmission electron microscopic analysis of ultrastructure. Cells were exposed for 24 hours to media alone (control) or NPs of CB and TiO_2 _and analyzed by TEM (control ×28000, CB ×28000 and TiO2 ×27000). Stars represent peripheral chromatin condensation, white arrow particle aggregates inside vesicles and grey arrow membrane blebbing. E) Time course analysis (0.5-24 h) of caspase-8 and caspase-3,-7 activation in CB and TiO_2 _NP treated cells (20 μg.cm^-2^). Cells were incubated with specific substrates for the respective caspases (Vybrant kit) and the % of caspase active cells was detected by flow cytometry. Data are represented as mean ± SD (n = 3). Representative results of at least three independent experiments. * statistically different from control p < 0.05 (two tailed).

Fluorescein diacetate/ethidium bromide (FDA/EtBr) double staining was used to differentiate between the apoptotic and necrotic cells. FDA/EtBr staining revealed a dose (8% and 10% increase of apoptotic cells from 20 μg.cm^-2 ^to 40 μg.cm^-2 ^for CB and TiO_2 _NPs respectively) and time dependent (starting from 4 hours) apoptotic cell death induction in 16HBE14o- cells by both types of NPs (Figure 2A, Additional file 1 figure S1). DNA fragmentation was detected by flow cytometry by measuring the sub-G1 peak in cell cycle analysis. A time course study of DNA fragmentation revealed that both types of NPs induce a significant increase in Sub-G1 peak from 4 hours of exposure (33 ± 2% and 26 ± 3% cells for CB and TiO_2 _NPs respectively) (Figure 2B). A significant decrease in forward scatter (FS) of the laser in flow cytometry, indicative of cell shrinkage, was observed after treatment with both NPs. A time course study revealed that exposure to both types of NPs cause a rapid decrease in cell size which is significant from 30 minutes and persists till 24 hours (11% decrease for CB and 22% for TiO2 NPs at 24 h) (Figure 2C). Ultra-structural analysis demonstrated that a significant proportion of cells exposed to NPs exhibit characteristic morphological features of apoptosis (membrane blebbing, formation of apoptotic bodies and chromatin condensation) (Figure 2D). These data were further strengthened by counting the number of cells exhibiting features of apoptosis on transmission electron microscopy (TEM) sections (data not shown). Caspase activation is taken as a putative molecular marker of apoptosis. Caspase assays indicated a significant activation of the initiator caspase (caspase-8) and executioner caspases (caspase-3 and -7) after treatment with both types of NPs (Figure 2E). A time course study revealed that the percentage of the caspase-8 active cells increases significantly after 1 hour of exposure to CB NPs (19 ± 2% of cells) and is maximum at 2 hours (23 ± 3% of cells). This activation persists till 24 hours, while caspase-3 and caspase-7 activation is maximum after 3 hours of exposure (27 ± 4% of positive cells). In case of TiO_2 _NPs, caspase-8 activation shows a similar time course as after CB NP exposures (22 ± 1% positive cells after 1 h) while caspase-3 and caspase-7 activation is maximum after 2 hours (32 ± 5% cells).

### Molecular Pathways of Cell Death Induction

**Figure 3:**
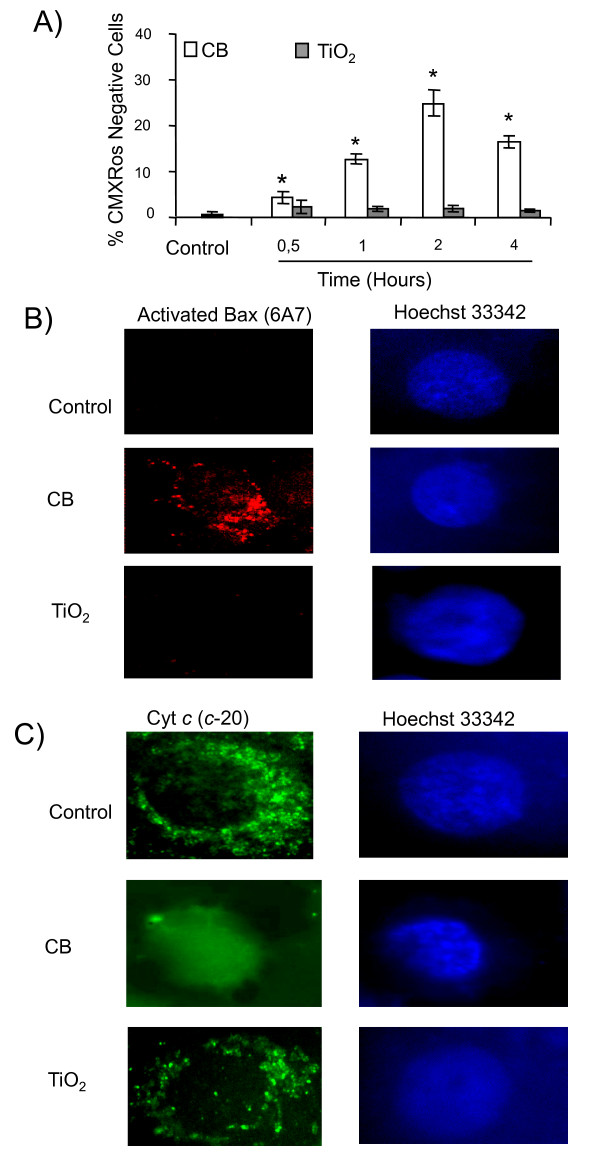
**Distinct apoptotic pathways in 16HBE14o- cells after CB and TiO_2 _NP exposure: role of mitochondrial pathway**. A) Loss of mitochondrial membrane potential. A time course study (0.5 -- 4 hours) after exposure to CB and TiO_2 _NPs (20 μg.cm^-2^). At the end of incubation cells were labeled with CMXRos fluorochromes (150 nM) for 20 minutes and % of CMXRos negative cells was determined by flow cytometry. Data are represented as mean ± SD (n = 3). Representative results of at least three independent experiments. * statistically different from control p < 0.05 (two tailed). C) Immunofluorescent detection of apoptotic proteins involved in mitochondrial pathway in 16HBE 14o- cells after 4 hours of NP exposure (20 μg.cm^-2^). Representative images of a cell undergoing apoptosis after treatment with CB and TiO_2 _NPs. Cells were stained with monoclonal antibodies against B) activated bax (6A7) C) cytochrome c (C-20). Nuclei were counterstained with Hoechst 33342. (×1000)

A time course study for the loss of mitochondrial membrane potential (ΔΨ_m_) using CMX Ros probe revealed that CB NPs cause a loss of ΔΨ_m _in a time dependent manner which is significant within 30 minutes (6 ± 1% of cells) and maximum after 2 hours of exposure (25 ± 2% of cells) whereas TiO_2 _NPs were unable to cause a loss of ΔΨ_m _(Figure 3A). Immunofluorescence staining for apoptotic proteins indicated activation of Bax and release of cytochrome *c *from mitochondria only in case of CB NPs (Figure 3B, 3C).

**Figure 4:**
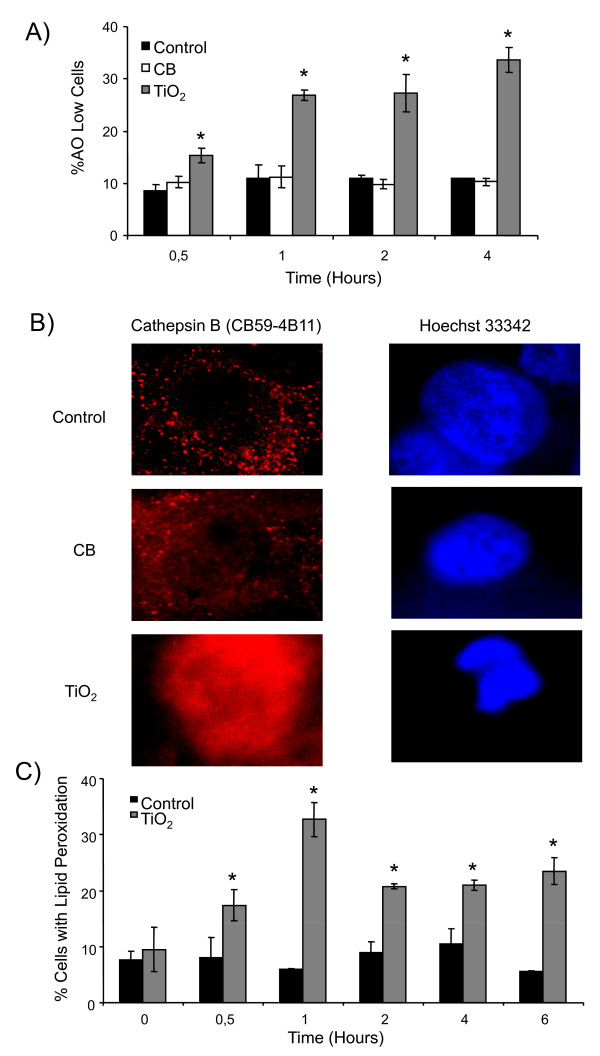
**Distinct apoptotic pathways in 16HBE14o- cells after CB and TiO_2 _NP exposure: role of lysosomal destabilization and lipid peroxidation**. A) Time course study (0.5-4 hours) of acridine orange loading of lysosomes by flow cytometry. Cells were preloaded with acridine orange (0.5 μg.mL^-1^) for 30 minutes, treated with CB or TiO_2 _at 20 μg.cm^-2 ^and analyzed by flow cytometry to determine the % of cells with a low AO staining. B) Immunofluorescent detection of release of lysosomal protease (cathepsin B) from destabilized lysosomes after 4 hours of exposure to NPs at 20 μg.cm^-2^. Cells were stained with monoclonal antibodies against cathepsin B (CB59-4B11). Nuclei were counterstained with Hoechst 33342. Representative images of a cell undergoing apoptosis after treatment with CB and TiO_2 _NPs (×1000). C) Induction of lipid peroxidation. Cell were preloaded with 10 μM Bodipy fluorophore for 30 minutes, exposed to NPs for 0-6 hours at 20 μg.cm^-2 ^and analysed by flow cytometry to determine the % of cells with lipid peroxidation. Data are represented as mean ± SD (n = 3). Representative result of three independent experiments. * statistically different from control p < 0.05 (two tailed).

Lysosomal destabilization and resulting release of proteases can play an important part in apoptosis. The destabilisation of lysosomal membranes was evaluated by acridine orange staining using flow cytometry. Acridine orange preferentially concentrates in the acidic organelles and a decrease in fluorescence occurs after membrane damage of these organelles. A time course study of AO staining revealed that only TiO_2 _NPs induce the destabilization of lysosomal membranes which is significant after 30 minutes (17 ± 1% of cells) and increases with time (32 ± 4% of cells at 4 hours) (Figure 4A). Lysosomal membrane destabilization leads to release of lysosomal proteases like cathepsins. We have demonstrated by immunofluorescence release of cathepsin B from lysosomes after treatment with TiO_2 _NPs whereas CB NPs had no effect (Figure 4B). Lipid peroxidation, which is also known to induce apoptosis, was evaluated by flow cytometry using bodipy 665/667 probe. We have demonstrated that TiO_2 _NPs induce a rapid and time dependent increase in lipid peroxidation which is maximum after 1 hour of exposure (33 ± 4% of cells) (Figure 4C). Unfortunately we were unable to measure lipid peroxidation for the CB NPs due to interference between the fluorochrome and NPs.

### Role of Oxidative Stress in apoptotic effects of NPs

**Figure 5:**
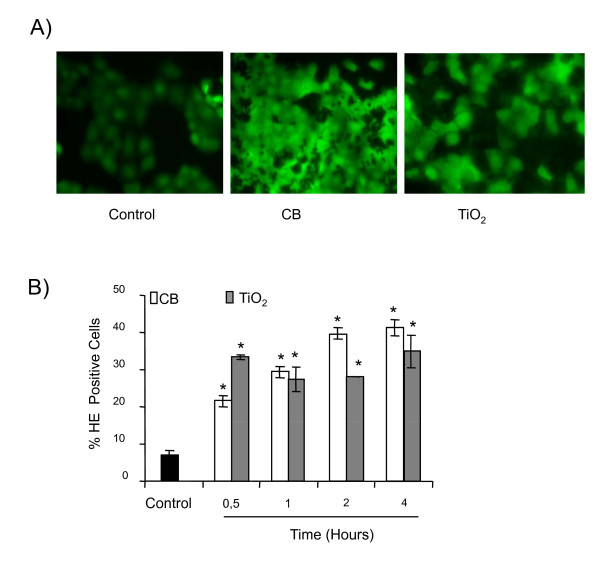
**Induction of oxidative stress in 16HBE14o- cells by CB and TiO_2 _NPs**. A) Cells were exposed to NPs (20 μg.cm^-2^) for 4 hours. At the end of exposure cells were loaded with DCFH-DA (40 μM) for 30 minutes and visualized under fluorescent microscopy (× 200). B) A time course study of HE staining (0.5 -- 4 hours) after exposure to CB and TiO_2 _NPs (20 μg.cm^-2^). At the end of incubation cells were labeled with HE fluorochrome (1 μM) for 20 minutes and analyzed by flow cytometry to determine the % of cells stained with HE. Data are represented as mean ± SD (n = 3). Representative result of three independent experiments. * statistically different from control p < 0.05 (two tailed).

As a putative role of oxidative stress has been postulated in the apoptosis, we further evaluated the abilities of NPs to cause oxidative stress in bronchial epithelial cells and studied the possible role of oxidative stress in apoptosis induction by these NPs. Figure 5A reveals an increased fluorescent staining of the cells due to DCFH-DA oxidation after exposure to CB and TiO_2 _NPs which is reduced in the presence of 1000 I.U. of PEG-catalase only in case of CB NPs (data not shown). A time course study was done to evaluate the ROS production by flow cytometry using HE probe. This study revealed that both CB and TiO_2 _NPs are able to significantly increase intracellular ROS in a time dependent manner (significant after 30 minutes of treatment) (Figure 5B).

**Figure 6:**
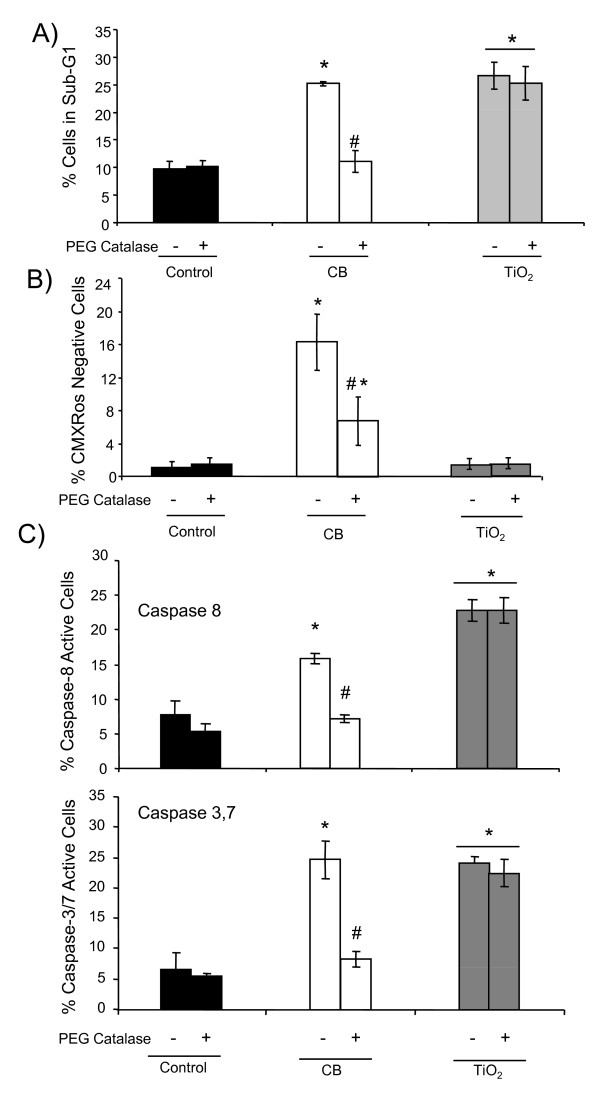
**Role of oxidative stress in the apoptosis induction by NPs in 16HBE 14o- cells**. Flow cytometry analysis after 4 hours of NP exposure (20 μg.cm^-2^) with or without PEG-catalase after 30 minutes of pretreatment with 1000 I U PEG-catalase A) DNA fragmentation (% of cells in the sub-G1 peak) B) loss of mitochondrial membrane potential (% of cells with low CMXRos staining) C) caspase activation (% of vibrant positive cells). Data are represented as mean ± SD (n = 3). Representative result of three independent experiments. * statistically different from control p < 0.05 (two tailed). # statistically different from particle treated group without catalase p < 0.05 (two tailed).

DNA fragmentation, loss of ΔΨ_m _and caspase activation are effectively modulated when cells were co-treated with 1000 I.U. of PEG-catalase only in case of CB NPs (Figure 6A, 6B, 6C) indicating the possible involvement of H_2_O_2 _production by these NPs.

### Confirmation of observed processes on normal human bronchial epithelial (NHBE) cells

**Figure 7:**
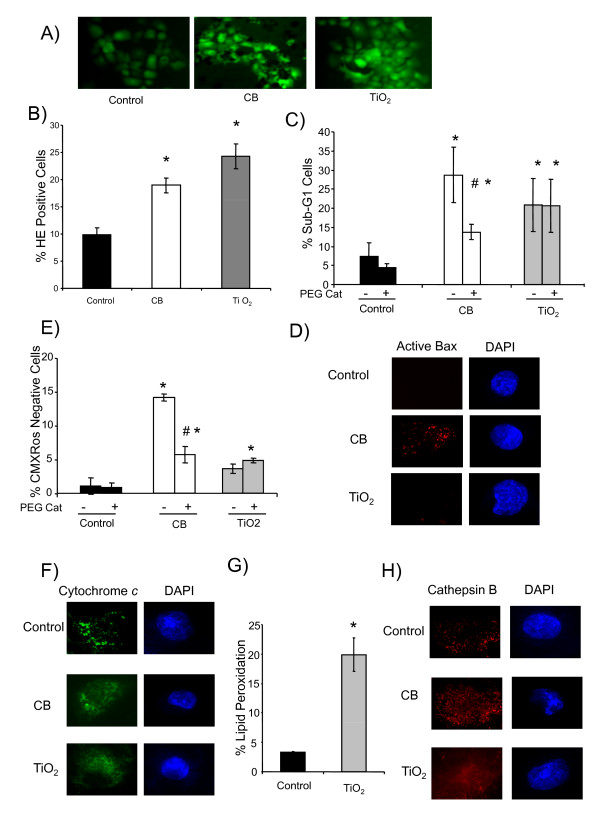
**Confirmation of distinct apoptotic pathways in normal human bronchial epithelial cells (NHBE cells) after CB and TiO_2 _NPs exposure at 20 μg.cm^-2 ^for 4 hours**. A) oxidative stress (DCFH-DA staining analyzed by fluorescent microscopy) (× 200). B) oxidative stress (% HE positive cells by flow cytometry) C) DNA fragmentation (% of cells in sub-G1 peak by flow cytometry), D) loss of mitochondrial membrane potential (% cells with low CMXRos staining by flow cytometry) E, F) immuno fluorescence for activated Bax (6A7) and cytochrome c (c-20). Nuclei were counterstained with DAPI. (×1000) Representative images of a cell undergoing apoptosis after treatment with CB and TiO_2 _NPs. G) lipid peroxidation (% of cells with high bodipy staining by flow cytometry) H) immuno fluorescence for cathepsin B (CB59-4B11). Nuclei were counterstained with DAPI. (×1000) Representative images of a cell undergoing apoptosis after treatment with CB and TiO_2 _NPs. For HE staining, DNA fragmentation and loss of mitochondrial membrane potential (MMP) NHBE cells were analyzed after 4 hours NP exposure with or without pre and cotreatment with 1000 I.U. pegylated catalase. Data are represented as mean ± SD (n = 3). Representative result of three independent experiments. * statistically different from control p < 0.05 (two tailed). # statistically different from particle treated group without catalase p < 0.05 (two tailed).

Finally the differences in the key steps in apoptotic pathways induced by CB and TiO_2 _NPs were confirmed on NHBE cells. Figure 7A shows that a treatment with CB and TiO_2 _NPs (4 hours exposure at 20 μg.cm^-2^) induces oxidative stress (DCFH-DA staining) in NHBE cells. ROS production was further quantified by HE staining and Figure 7B shows that both NPs significantly increase the percentage of HE positive cells. DNA fragmentation (Sub-G1 peak) assay indicated that both NPs induce apoptosis and PEG-catalase was effective only in case of CB NPs (Figure 7C). We further evaluated the involvement of mitochondrial events in apoptosis induction by these NPs. A significant loss of ΔΨ_m _was only observed in case of CB NPs which was effectively modulated in the presence of PEG-catalase (Figure 7D). Moreover, immunostaining showed that CB NPs induce the activation of Bax and release of cytochrome *c *from mitochondria (Figure 7E, 7F). Lipid peroxidation induction was observed in case of TiO_2 _NPs (Figure 7G) and release of lysosomal protease (immunostaining of cathepsin B) was only observed in case of TiO_2 _NPs (Figure 7H).

## Discussion

NPs pose an emergent challenge to toxicologists as they are gaining diversified utilizations in many fields of life. A detailed description of molecular mechanisms induced by these NPs and their risk assessment is clearly warranted. This experimentation aimed at deciphering the mechanisms underlying the cytotoxic effects of CB and TiO_2 _NPs. We have previously shown that these NPs are capable of inducing cytotoxicity in bronchial epithelial cells[21]. There have been a few studies which attributed apoptosis as the basic phenomenon for cytotoxic effects of different types of NPs but these observations were mostly made on the basis of caspase activation or annexin V staining for apoptosis detection and did not address the molecular pathways[7,22,23]. A recent article by Pan and colleagues describes that 1.4 nm gold NPs induce necrosis by oxidative stress and mitochondrial damage in Hela cells[24]. A deeper insight into the signaling pathways triggered by NP exposures in their main target cells will help in identifying the potential diseases/disorders which may occur after NP exposures and might also help in understanding the contribution of NPs in the pathogenesis of these disorders. In vitro studies are preferred for the comprehension of molecular mechanisms and signaling pathways and it has been shown that *in vitro *doses of 20 μg.cm^-2 ^represent exposures of high risk individuals (asthmatics etc) to ambient levels of atmospheric particles [25,26].

Detailed physico-chemical characterization of both types of NPs was done before cell culture exposures. In cell culture medium, the surface charge on the NPs measured by the zeta potential is less negative than in water. This charge reduction is caused by the compensation of negative charges due to the presence of inorganic cations contained in the physiological media[27]. These cations are attracted to the negatively charged surface of the NPs. A less negative zeta potential favours particle aggregation, since the contribution of electrostatic repulsion is decreased[28]. We observed indeed that in physiological media, the hydrodynamic particle diameter is larger than in water as a result of this enhanced aggregation. Inside the human respiratory tract particles may also become aggregated as soon as they come in contact with the surfactant and mucous covering the pulmonary cells. The observation of cytotoxic effects from the aggregated NPs in the cell culture medium is not uncommon[29]. We have already shown that, even when in the form of aggregates, the cytotoxic, pro-inflammatory and oxidative effects of NPs depend upon the primary particle size/surface area[21]. We observed a few nanometer differences in the primary particle diameters we measured by TEM and data provided by the manufacturer (Table 1). This may be due to different methodologies used but these were not communicated by the manufacturer. Furthermore, we measured the diameters after suspending NPs in the cell culture medium, which might lead to coating of NPs. Nevertheless, this observation indicates the need to determine nanomaterial physicochemical characteristics under treatment conditions before experimentation.

Peripheral chromatin condensation, membrane blebbing and formation of apoptotic bodies is classical for apoptosis[30]. Recently similar ultrastructural features have been described in pulmonary epithelial cells of NP exposed factory workers[31]. TEM analysis confirmed the presence of these typical morphological features of apoptosis whereas necrosis was not observed at low doses. It is interesting to note that at least two to three times more doses of atmospheric particles are required to induce similar apoptotic effects (data not shown). Therefore we compared the cytotoxicity of the NPs used in this study with larger sized CB (21 nm and 95 nm) and TiO_2 _(50 nm) and cytotoxicity increases with decreasing particle size (CB13 nm> CB 21 nm >CB 95 nm for CB and TiO_2 _15 nm>TiO_2 _50 nm TiO_2 _NPs respectively)[21]. The morphological characterization of apoptotic cell death was confirmed with cytometric observations of cells with reduced size. The decrease in cell size (apoptotic volume decrease) is considered as a hallmark of apoptotic cell death and plays an important role in regulating the activity of apoptotic nucleases and activation of caspases[32]. Our observation of caspase activation by CB and TiO_2 _NPs is in agreement with other studies showing recently the activation of caspase-3 in the apoptotic events of CB and TiO_2 _NPs[22,33]. We have shown here that NP exposure leads to activation of both initiator (C-8) and executioner caspases (C-3/-7). Caspase-8 activation typically occurs in death receptor signaling pathway of apoptosis induction but recently it has been shown that alternative activation systems also exist [34-36]. Indeed, we did not observe the activation of Fas receptor after the treatment with NPs (data not shown). Furthermore, we have shown here that caspase 8 activation leads to the activation of caspase-3/-7 as its inhibition by a specific inhibitor (z-IETD-fmk) decreases caspase-3/-7 activation (Additional file 1 figure S2). Caspases have been shown to be sensitive to the redox status of the cells [16,37,38] and we have also shown a time dependent induction of oxidative stress by both types of NPs. Activation of executioner caspases (C-3/-7) leads to proteolysis and fragmentation of DNA which after fixation with permeablizing fixatives like methanol/ethanol leaks out of the cell and hypodiploid cells are detected as a subG1-peak in cell cycle analysis using PI incorporation. We have shown here that both CB and TiO_2 _NPs exposure leads to fragmentation of DNA and appearance of this sub-G1 peak occurs after the activation of caspases confirming the role of caspases in the fragmentation of the DNA.

Interestingly, after treatment with NPs a different outcome was observed for CB and TiO_2 _NPs in terms of loss of ΔΨ_m _which is only induced by CB. This clearly points out that different mechanisms of cell death induction can be triggered by different types of NPs according to their chemical nature. Loss of ΔΨ_m _occurs due to a variety of factors including mitochondrial outer membrane permeabilization (MOMP) and ROS. We have shown by the use of PEG- catalase that the CB NPs induced perturbation in mitochondrial potential, caspase activation and DNA fragmentation is dependent on the oxidative stress (H_2_O_2 _production) induced by these NPs whereas PEG-catalase could not protect against apoptosis induction by TiO_2 _NPs. Catalase is an antioxidant enzyme which specifically scavenges H_2_O_2 _and PEG modification facilitates intracellular uptake of catalase, elevates intracellular activity and augments cellular resistance to oxidative stress as shown for endothelial cells[39]. Moreover, heat inactivation of the PEG-catalase leads to loss of its protective effect indicating that the protection is due to its enzymatic activity and not due to non-specific coating on the particles (Additional file 1 figures S3). MOMP is considered as a point of no return of the programmed cell death process conducting to the release of pro-apoptotic cytochrome *c*, apoptosome formation and caspase activation [40-42]. We observed activation of Bax and release of cytochrome c only in case of CB NP exposure. In the presence of all these findings, we are convinced of the involvement of the mitochondrial pathway and oxidative stress (H_2_O_2_) only in case of apoptotic cell death induced by CB NPs.

To understand the initial phase of apoptosis induction in the case of TiO_2 _NPs we evaluated the contribution of the lysosomal compartment in the cell death pathways of these NPs. Lysosomal destabilization leads to release of lysosomal proteases particularly cathepsin B which participate in the final outcome of apoptotic phenomenon by either directly causing proteolysis or by activating other proteases like caspases [43-46] as leaked cathepsins in the vicinity of lysosomes are more likely to cleave procaspase-8[45,46]. Release of lysosomal proteases has been shown to be involved in cell death induction by a variety of stimuli (ROS, drugs, TNF alpha) [44,47-49]including silica NPs[50]. We observed a significant lysosomal destabilization only in case of TiO_2 _NPs. We have previously reported that NPs are internalized as aggregates which are essentially taken up in the phagosomes/phagolysosomes[21]. We analyzed the internalization of these NPs by flow cytometry and TEM and observed that these are internalized in a dose dependent manner in 16HBE cells. This fact points towards the possibility that TiO_2 _NPs may induce lysosomal damage during the endocytic uptake process leading to release of cathepsins. ROS are one of the variety of factors which cause lysosomal destabilization[51,52]. In addition to oxidative stress, we also observed a dose dependent lipid peroxidation by TiO_2 _NPs. These results are in agreement with previous studies which described oxidative DNA damage and lipid peroxidation in BEAS-2B cells without photo-activation of TiO_2 _NPs[19]. It has been postulated that ROS production (specifically hydroxyl radical production) can occur at the surface of TiO_2 _NPs even in the absence of UV illumination or sun light exposure [53]and it is a proven fact that hydroxyl radicals are principal agents which lead to lipid peroxidation. This may explain the different apoptotic pathways observed for TiO_2 _and CB NPs which did not induce lysosomal destabilisation. It is noteworthy that lipid peroxidation has also been shown to activate caspases in a cellular redox status depending manner[54]. Taken together, these data strongly suggest the contribution of lysosomes and lipid peroxidation in the cell death induction by TiO_2 _NPs. All these mechanisms were further confirmed in normal human bronchial epithelial cells (NHBE).

**Figure 8:**
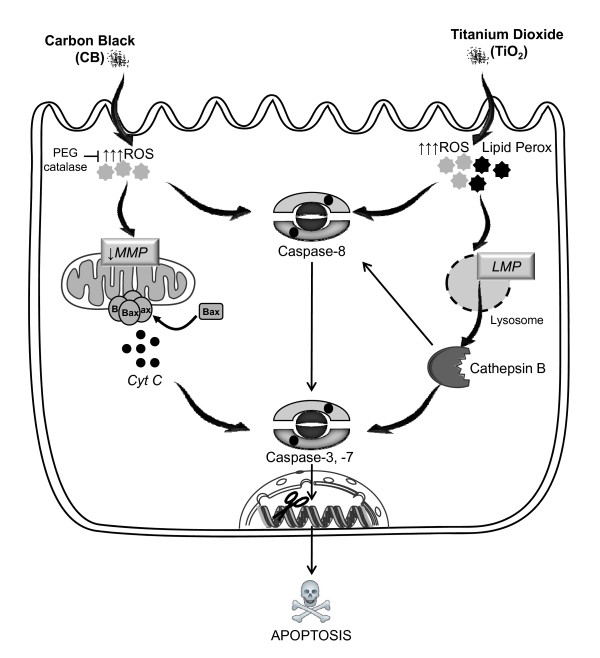
**Schema of the hypothetic pathways of cell death induction by CB and TiO_2 _NPs in bronchial epithelial cells**. CB NP induce apoptosis by a ROS dependent mitochondrial pathway involving loss of MMP, activation of bax and release of cytochrome c resulting in activation of caspases and subsequent DNA fragmentation. TiO_2_NPs induce cell death through lipid peroxidation and lysosomal membrane destabilization leading to cathepsin B release and subsequent activation of caspases and final apoptotic events. Modulation of oxidative stress by PEG catalase prevents cell death by blocking downstream events only in case of CB NPs. (Image drawn in part using Servier medical art)

Thus we conclude that the CB and TiO_2 _NPs induce a rapid apoptosis in bronchial epithelial cells and initial phase of cell death induction is different in both cases. In Figure 8, we have represented a schematic diagram of pathways involved in the apoptosis induction by both CB and TiO_2 _NPs. In case of CB a decrease in ΔΨ_m _and oxidative stress could already be observed after 30 minutes of exposure while caspase-3 activation is significantly increased from 2 hours and subG1 peak only after four hours. Apoptotic events depend on H_2_O_2 _formation in case of CB NPs as they could be effectively modulated by using PEG-catalase. This implies that initial production of ROS leads to loss of ΔΨ_m _causing subsequent release of cytochrome *c *and activation of C-3, C-7 which leads to DNA fragmentation. In case of TiO_2 _NPs, initial ROS production, lipid peroxidation and lysosomal membrane destabilization (all significantly induced from 30 minutes of exposure) lead to release of cathepsin B and activation of caspase-8 after 1 hour and caspase-3/7 after 2 hours resulting in DNA fragmentation which is only observed after 4 hours.

## Conclusion

In conclusion we have elucidated different molecular pathways that mediate the apoptotic effects of NPs in bronchial epithelial cells (16HBE14o- and NHBE cells). Indeed, the initial phase of cell death induction varies according to the chemical nature of NPs. In case of CB NPs the production of ROS is implicated in the downstream mitochondrial events whereas, in case of TiO_2 _NPs the initial lysosomal destabilization and lipid peroxidation is involved in the apoptosis induction. These results will help in understanding the possible contribution of NPs in the pathogenesis of those disorders in which apoptosis plays an important role and in a longer run will assist in the development of strategies to counteract the possible adverse health effects of NPs. Our results clearly point towards the need of meticulous understanding of molecular events occurring after NP exposures and not just looking at the final outcome as the pathways may differ for diverse NPs. Indeed, the presence of distinct pathways raises the possibilities of observing different effects of the NPs at non cytotoxic concentrations which may induce other pathophysiological phenomenon.

## Materials and methods

All the chemicals were purchased from Invitrogen (Cergy-Pontoise, France) unless otherwise specified.

### Nanoparticles

CB and TiO_2 _NPs were purchased from Evonik Industries/Degussa (Frankfurt, Germany) and Sigma-Aldrich (Saint Quentin Fallavier, France) respectively. CB NPs were produced through a 'gas-russ' process and post-treated by the manufacturer with oxidants (oxidized surface). TiO_2 _NPs (99.7% pure anatase, X-ray diffraction analysis) utilized were without any surface treatment. No surfactant was used to treat NP surfaces. Aluminium racks were used to overcome the disturbances in weighing caused by high electrostatic potential of NPs. Stock suspensions of the NPs were made at a concentration of 2 mg.ml^-1 ^in DMEM/F12 (Dulbecco Modified Eagle Medium Nutrient Mix F-12) cell culture media containing 15 mM HEPES, sonicated for 3 mins at 60 W with the help of an induced ultrasonic probe (Ultrasonic Processor, Fisher Bioblock Scientific, Illkirch France) and stored at -20°C till further use. NPs were sonicated three times for 20 s at 60 W within 5 minutes prior to dilution in cell culture media. For most of the experiments NP exposure was done at 20 μg.cm^-2 ^corresponding to 108 μg.mL^-1^.

### Nanoparticle Characterization

#### Dynamic Light Scattering and Zeta Potential Measurement

NP stock suspensions (2 mg.ml^-1^) were prepared and sonicated for 60 seconds at 60 W (Bioblock ultrasonic processor 75038, Bioblock Scientific, IIIkrich, France)). Stock suspensions were diluted in water, PBS or DMEM F-12 cell culture medium. Dynamic light scattering experiments were performed with Brookhaven 90 Plus (Brookhaven Intstruments Co, New York, USA). Scattered laser light (659 nm, 15 mW) was detected under an angle of 90°. The fluctuations in the scattered laser light intensity are correlated by 200 channels between 0.1 μs and 0.1 s. Correlation functions were analyzed with Igor Pro 6.02A using the Clementine package for modeling of Decay kinetics using Maximum Entropy method resulting in intensity weighted distribution functions versus decay times. By converting the decay times with instrument parameters and Stokes-Einstein law to hydrodynamic diameters, an intensity weighted size distribution was obtained. Peak positions and widths were fitted with a lognormal function to obtain the average diameter of each resolved NP population. Zeta potential was measured with Brookhaven ZetaPals option (Brookhaven Intstruments Co, New York, USA). The ELS (electrophoretic light scattering) technique employed is based on reference beam (659 nm, 35 mW), modulated optics and a dip-in electrode system. The shift in frequency, ω_s _of scattered light from a charged particle moving in an electric field is related to the electrophoretic mobility of the particle. The Smoluchowski limit relates the electrophoretic mobility, μ_e _to the zeta potential: μ_e _= ες/η where ε = Permittivity of Liquid, ς = Zeta Potential and η = Viscosity of Liquid

Further details on the physicochemical characteristics of these NPs have already been published using Scanning Electron Microscopy, turbidimetry and TEM [21,55] and surface functional group analysis[56]. Further details on CB NPs are available through Degussa (Pigmentrusse/pigment blacks Technische Daten Europa/Technical Data Europe, Degussa AG, Advanced Fillers & Pigments, 2006).

### Cell Culture Conditions

#### 16HBE14o- cells

A SV40 large T- antigen transformed bronchial epithelial cell line (16HBE14o-) which retains undifferentiated epithelial morphology and functions was utilized in this study[57]. These cells were generously provided by Dr. DC. Gruenert (Medical Research Facility, California Pacific Medical Centre, San Francisco, CA, USA). 16HBE14o- cell line is produced by transformation with a construct encoding an origin-defective simian virus SV40 large T, known to inhibit the E2F1 binding activity of pRb and inactivate the tumor suppressor p53[57,58]. There are precedents in the literature about the use of this cell line to study the mechanisms of apoptosis induction by various agents like doxycycline, retinoic acid, mustard and oxidative stress [58-61]. All experiments were performed using cells between passages 35-45. Cells were grown in DMEM/F12 cell culture media supplemented with penicillin (Sigma-Aldrich, 100 μg/mL), streptomycin (Sigma-Aldrich,100 μg/mL), fungizone (Sigma-Aldrich,1 μg/mL), glutamine (Sigma-Aldrich, 0.292 mg/mL) and Ultroser G (UG) 2% (Biosepra Cergy Pontoise France). Cells were cultivated on the plastic material (Costar, VWR Fontany- Sous- Bois France) after coating with type I collagen (Sigma-Aldrich, 4 μg.cm^-2^) and maintained in 5% CO_2 _environment at 37°C temperature in an incubator. In most experiments, except otherwise specified, cells were seeded in 12 well plates at a density of 10,000 cells.cm^-2 ^and after 48 h of growth, they were grown for further 24 h in UG free media before treatment with NPs. For immunofluorescence analysis cells were seeded in Lab-Tek^® ^culture plates. For experiments with poly ethylene glycol coated catalase (PEG-catalase, Sigma Aldrich, PEG mol. wt 5000 ~50% protein with ~40 mol PEG per mol of protein) cells were incubated for 30 minutes with 1000 I.U. of enzyme before adding NPs and then exposed to NPs in the presence of this antioxidant.

#### Normal Human Bronchial Epithelial Cells (NHBE)

NHBE cells were purchased from Lonza (Walkersville, MD USA). These cells were cultivated in BEBM media supplemented with 5 μg/mL insulin, 0.5 ng/mL hEGF, 0.5 μg/mL hydrocortisone, 0.5 μg/mL epinephrine, 50 μg/mL gentamycin, 50 μg/mL amphoteracin B, 10 μg/mL transferrin, 6.5 ng/mL triidotyronin, 0.13 mg/mL bovine pituitary extract (all supplied by Lonza). Cells were cultivated on non coated plastic material under similar conditions as mentioned for the 16HBE cell line. After 72 h of growth cells were grown in media without growth factors for further 24 h. Cells were treated in media without growth factors following the same protocol as described for 16HBE cells. All the experiments were performed between passage 1-5.

### Ultra Structural Analysis

Morphological features of cell death induced by CB and TiO_2 _NPs were studied using TEM. Cells were treated with NPs (5 μg.cm^-2^) for 24 h. Fixation and Epon embedding of cells was performed as described elsewhere[62]. Ultrafine sections (60 nm thick) were collected on copper grids and studied using a JEOL 1200 EXII microscope fitted with an energy dispersive spectrometer (OXFORD LINK ISIS 300).

### Fluorescein Diacetate and Ethidium Bromide Staining

Discrimination between live, apoptotic and necrotic cells was made by fluorescein diacetate (FDA, Sigma-Aldrich, 1 μg/ml in DMEMF/12) and ethidium bromide (EtBr, Sigma Aldrich, 10 μg/ml in DMEMF/12) staining as previously described[63]. Cells were differentiated into healthy (green fluorescent cells without any nuclear staining), apoptotic (condensed or fragmented orange red nucleus) or necrotic (orange red 'apparently normal' or patchy nucleus). At least 600 cells were observed per treatment condition on an epifluorescence microscope.

### Intracellular Reactive Oxygen Species (ROS) Production

ROS production was evaluated by hydroethidine (HE) probe with slight modifications to Rothe et al[64]. Cell were treated with CB and TiO_2 _NPs for the desired time, supernatants were harvested and added to the tyripsinated cells. After addition of 10% FCS, samples were centrifuged for 10 minutes at 400 g. Cells were resuspended in cell culture media containing HE at 1 μM final concentration and incubated at 37°C for 20 min. Analysis was performed with the help of CyAn ADP LX DakoCytomentation equipment at 488 nm excitation and 615 nm emission wavelengths. After eliminating cell debris at least 10000 cells were analyzed to determine the percentage of HE positive cells.

ROS production was also evaluated by DCFH-DA staining as described previously[33]. Briefly, at the end of 4 hours of exposure to NPs cells were treated with 40 μM 2',7'-diclorodihydrofluorescein diacetate (DCFH-DA) (Sigma Aldrich) for 30 minutes and cells were imaged by using a fluorescence microscope (Nikon Optiphot). It was confirmed before experimentation that particles themselves did not cause oxidation of the fluorochrome.

### DNA Fragmentation Analysis

After trypsination cells were added to the supernatants, centrifuged at 100 g for and overnight fixed in 1 ml methanol at -20°C. Cells were washed with PBS two times and then suspended in PBS containing 250 μg/ml RNase (Sigma Aldrich). After 20 minutes, PI (20 μg/ml) (Sigma Aldrich) was added and cells were further incubated for 1 hour at room temperature. After the incubation cells were analyzed with the help of CyAn ADP LX DakoCytomentation equipment at 488 nm excitation and 615 emission wavelength. At least 10000 cells were analyzed to determine the percentage of cells in the Sub-G1 region.

### Loss of Mitochondrial Membrane Potential

Mitochondrial membrane potential dynamics were evaluated by measuring ΔΨ_m _with mitochondrial selective probe MitoTracker^® ^red (CMXRos) according to manufacturers recommendations. Cells were prepared as described above and after eliminating debris analysis of at least 10000 cells was performed on CyAn ADPLX Dako Cytomentation equipment using 488 nm excitation and 615 nm emission wavelengths.

### Lipid Peroxidation

Lipid peroxidation was evaluated by Bodipy 665/667 fluorophore (Molecular Probes) which is a non polar derivative of Bodipy probe. Oxidation of this lipophillic fluorophore occurs in the presence of peroxyradicals[65]. Cells were incubated with 10 μM probe for 30 minutes and then treated with NPs and processed for cytometry analysis as described above. The loss in red fluorescence was measured using CyAn ADPLX DakoCytomentation equipment using 488 nm excitation and 615 nm emission wavelengths. After eliminating debris analysis of at least 10000 cells was performed to determine the % of cells.

### Caspase assay

After exposure to NPs, cells were incubated with specific caspase substrates or inhibitors for caspases 3,7 and 8 (Vybrant^® ^FAM caspase assay kits, Molecular Probes) according to manufacturers recommendations and at least 10 000 cells were analyzed by flow cytometry (CyAn LX Dako Cytomentation equipment) using 488 nm excitation and 530 nm emission wavelengths. For experiments using inhibitors for caspase 8 (Z-IETD-FMK) (Bachem Biochimie, France) cells were incubated with 50 μM Z-IETD for 30 minutes before adding NPs.

### Immunofluoresence for Apoptotic Proteins

Cells were fixed in paraformaldehyde (Sigma-Aldrich, 0.4% in PBS) for 30 minutes at 25°C and incubated for 10 minutes with NH_4_Cl (Sigma-Aldrich, 50 mM) before permeabilization in PBS 0.05% Tween 20 (Sigma-Aldrich, 0.05%). After saturation in PBS, Tween 20 (0.01%), BSA (Bovine Serum Albumin, Sigma-Aldrich, 3%) cells were incubated for 30 minutes with mouse monoclonal anti-Bax 6A7 (1:100) (Sigma-Aldrich), goat polyclonal anti-cytochrome *c *C-20 (Santa Cruz Biotechnology Inc, 1:100) or mouse monoclonal anti-cathepsin B(CB59-4B11, Sigma-Aldrich) (1:100) antibodies in PBS containing 0.01% Tween 20 and 3% BSA. Secondary anti mouse antibodies Alexa fluor 546-IgG or FITC-IgG (1:100) (Dako, Trappes, France) were diluted in PBS containing 0.01% Tween 20 and 3% BSA at 1:500 and 1:100 respectively and incubated for 30 minutes. Nuclei were stained with Hoechest 33342 (2 μM) for 5 minutes. Cells were mounted in 0.1 M Tris (Sigma-Aldrich, pH 8.5) containing 10% mowiol (Sigma-Aldrich), 25% glycerol (Sigma-Aldrich) and 2.5% 1,4-DiAzaBiCyclo(2.2.)Octane (DAPCO, Sigma-Aldrich) and examined under a Leica SP2 confocal microscope. Image treatment was done on Image J software (Image J 1.42 NIH, USA).

### Acridine Orange Staining

Lysosomal permeability was evaluated by acridine orange staining (AO) as described previously[48,50] using flow cytometry. AO in an acidic environment, as encountered in lysosomes, emits red fluorescence when excited by blue light. The intensity of red fluorescence is a direct indicator of lysosomal concentration and its decrease reflects lysosomal membrane damage[50,66]. Briefly, cells were preloaded with 0.5 μg.mL^-1 ^AO in DMEM/F12 media for 30 minutes at 37°C, rinsed at least three times with DMEM-F12 media, and exposed to NPs for 4 hours. After incubation cells were trypsinated and added to supernatants. After eliminating debris at least 10 000 cells were analysed by a CyAn ADP LX flow cytometer using 488 nm excitation and 620 nm emission wavelengths.

### Cell Size Analysis

Cytometry data obtained from all above mentioned methods was subjected to cell size analysis (forward scatter (FS) of laser). Relative cell size (taking control as 100%) was calculated.

### Statistical Analysis

Every experiment was repeated at least three times with triplicate of each condition. Data are represented as means ± SD and were analyzed on a commercially available software GRAPHPAD (Graphpad Prism 4.01, Graphpad Software Inc, San Diego, USA) by analysis of variance (ANOVA) followed by Bonferroni test for multiple comparisons with p < 0.05 (two tailed) considered as significant.

## Competing interests

The authors declare that they have no competing interests.

## Authors' contributions

SB and SH contributed in study design, did the experimental work, analyzed data and wrote the manuscript. LT and JM did the particle characterization. CB and JF helped in TEM analysis. IF helped in experimental work and reviewed the manuscript. FM, AB, KA critically reviewed the manuscript and gave intellectual input. All the authors have read and approved final manuscript.

## Supplementary Material

Additional file 1 **Supplementary figures**. **Figure S1 **- Time course analysis of FDA/BET staining in CB and TiO_2 _NP treated 16HBE14o- cells. **Figure S2 **- Cross talk between caspase-8 and caspase-3/-7 in CB and TiO_2 _NP treated 16HBE14o- cells. **Figure S3 **- Effect of heat inactivation of PEG-catalase on mitochondrial membrane potential in CB and TiO_2 _NP treated 16HBE14o- cells. Click here for file
