# Supplementary material for: Carbon black and titanium dioxide nanoparticles elicit distinct apoptotic pathways in bronchial epithelial cells
Source: Part Fibre Toxicol. 2010 Apr 16;7:10. doi: 10.1186/1743-8977-7-10 (PMC2873464; doi:10.1186/1743-8977-7-10)
Supplement: Additional file 1 — Supplementary figures. Figure S1 - Time course analysis of FDA/BET staining in CB and TiO2 NP treated 16HBE14o- cells. Figure S2 - Cross talk between caspase-8 and caspase-3/-7 in CB and TiO2 NP treated 16HBE14o- cells. Figure S3 - Effect of heat inactivation of PEG-catalase on mitochondrial membrane potential in CB and TiO2 NP treated 16HBE14o- cells. [file 1743-8977-7-10-S1.DOC]

**Additional File 1 Hussain et al**

Additional file1 figure S1

Additional file1 figure S2

Additional file1 figure S3

**Legends:**

**Additional file 1 figure S1.**

Characterization of cell death in 16HBE14o- cells by fluorescent microscopy.

A time course study (0.5- 24 hours) of FDA (fluorescein diacetate) and EtBr (ethidium bromide) staining after 20µg.cm^-2^NPs exposure. Data are represented as mean ± SD of at least three independent experiments. * statistically different from control p< 0.05 (two tailed).

**Additional file 1 figure S2.**

Caspase activation in 16HBE14o- cells.

Activity of caspase-3/-7 either in the presence or absence of caspase 8 inhibitor (IETD). Cells were pretreated with IETD for 30 minutes and then exposed to NPs for 4hours (20µg.cm^-2^). Data are represented as mean ± SD of at least three independent experiments. * statistically different from control # statistically different from particle treated group without IETD, p< 0.05 (two tailed).

**Additional file 1 figure S3.**

Effect of heat inactivation of PEG-catalase on mitochondrial membrane potential in 16HBE14o- cells.

Cells were pre-incubated with either PEG-Cat N (PEG catalase without heat inactivation) or PEG-Cat H (PEG catalase after heat inactivation; 100°C 1 minute) and exposed to NPs of CB for 4 hours (20µg.cm^-2^) in the presence of antioxidant. Cells were harvested and percentage of CMXRos negative cells was estimated by flow cytometry. Data are represented as mean ± SD. * statistically different from control p< 0.05 (two tailed) # statistically different from NP treated cells with out antioxidant pretreatment.
